# Fear of death and the pandemic

**DOI:** 10.1192/j.eurpsy.2022.1392

**Published:** 2022-09-01

**Authors:** A.H.I. Abu Shehab, A.V. Gurita, L. Luca, N. Isabela, M. Terpan, A. Ciubara

**Affiliations:** 1 ’’Elisabeta Doamna’’ Psychiatry Hospital of Galati, Psychiatry Department, Galati, Romania; 2 ’Elisabeta Doamna’’ Psychiatry Hospital of Galati, Psychiatry Department, Galati, Romania; 3 University of Medicine and Pharmacy “Grigore T. Popa”, Psychology, Iasi, Romania; 4 ’Dunerea de jos’’ University of Galati, Psychiatry Department, Galati, Romania

**Keywords:** pandemic, Covid-19, fear

## Abstract

**Introduction:**

Looking at the vast majority of mental disorders in the last year, we noticed that most of them were closely related to this feeling of fear but also to the restrictive measures that appeared with the pandemic. Exposure for a period of more than a year to this mental stress has led to the appearance of a large number of psychiatric patients, especially those who have undergone SARS-CoV-2 infection or who have had close people infected, some of whom have even died.

**Objectives:**

In this paper I will highlight the post traumatic consequences in patients who have gone through the disease.

**Methods:**

To complete this work I used medical articles, studies, and specialized information on the subject.

**Results:**

Patients who have gone through the disease developed sleeping problems, phobias, various anxiety and delusional disorders.

**Conclusions:**

These conditions create the need for a multidisciplinary approach among this particular category of patients.

**Disclosure:**

No significant relationships.

